# Functional transcriptome analyses of *Drosophila suzukii* midgut reveal mating-dependent reproductive plasticity in females

**DOI:** 10.1186/s12864-022-08962-2

**Published:** 2022-10-25

**Authors:** Shisi Xing, Dan Deng, Wen wen, Wei Peng

**Affiliations:** Hunan Provincial Key Laboratory of Animal Intestinal Function and Regulation, State Key Laboratory of Developmental Biology of Freshwater Fish, HunanInternational Joint Laboratory of Animal Intestinal Ecology and Health, Hunan Normal University, Changsha, 410081 China

**Keywords:** Spotted wing drosophila, Intestinal RNA-seq, Female mating status, Reproductive plasticity, Gene expression

## Abstract

**Background:**

Insect females undergo a huge transition in energy homeostasis after mating to compensate for nutrient investment during reproduction. To manage with this shift in metabolism, mated females experience extensive morphological, behavioral and physiological changes, including increased food intake and altered digestive processes. However, the mechanisms by which the digestive system responds to mating in females remain barely characterized. Here we performed transcriptomic analysis of the main digestive organ, the midgut, to investigate how gene expression varies with female mating status in *Drosophila suzukii*, a destructive and invasive soft fruit pest.

**Results:**

We sequenced 15,275 unique genes with an average length of 1,467 bp. In total, 652 differentially expressed genes (DEGs) were detected between virgin and mated *D. suzukii* female midgut libraries. The DEGs were functionally annotated utilizing the GO and KEGG pathway annotation methods. Our results showed that the major GO terms associated with the DEGs from the virgin versus mated female midgut were largely appointed to the metabolic process, response to stimulus and immune system process. We obtained a mass of protein and lipid metabolism genes which were up-regulated and carbohydrate metabolism and immune-related genes which were down-regulated at different time points after mating in female midgut by qRT-PCR. These changes in metabolism and immunity may help supply the female with the nutrients and energy required to sustain egg production.

**Conclusion:**

Our study characterizes the transcriptional mechanisms driven by mating in the *D. suzukii* female midgut. Identification and characterization of the DEGs between virgin and mated females midgut will not only be crucial to better understand molecular research related to intestine plasticity during reproduction, but may also provide abundant target genes for the development of effective and ecofriendly pest control strategies against this economically important species.

**Supplementary Information:**

The online version contains supplementary material available at 10.1186/s12864-022-08962-2.

## Introduction

Reproduction is an energetically costly process which induces numerous physiological and functional adaptations in the gastrointestinal tract during pregnancy and lactation in multifarious species [1, 2]. Female individuals invest more energy and resources than male individuals into reproduction, and restructure energy balance to maximise their reproductive success. These shifts fulfill sufficient nutrient intake to the increasing energy demands in females [2]. In insects, such as the fruit fly *Drosophila melanogaster*, dietary protein is required for yolk protein synthesis, and changing female’ s protein intake can affect her fecundity [3–9]. Moreover, mated females enhance feeding and consume energy and protein rich diets preferentially to support the metabolic needs of oviposition [10–15]. As the major place of digestion and nutrient absorption, the female midgut is a crucial regulator of alterations in post-mating energy balance, and signals between midgut and ovary are critical for raising egg production after mating [16–19]. The adult *D. melanogaster* intestine is a plastic organ, and the female midgut undergoes striking remodeling in size and physiology by stimulating intestinal stem cell (ISC) driven epithelial expansion in responding to mating [17, 20–22].

The *Drosophila* midgut is sexually dimorphic which is reflected in the differences of physiology and gene expression [17, 20, 23, 24]. Cell division-related processes genes and carbohydrate metabolism genes are abundantly expressed in females and males respectively [23]. Compared to the males, ISC proliferation is higher in virgin females and this sex difference is further improved by mating, since ISC proliferation is significantly higher in mated females than in virgin females [17, 23, 25]. Furthermore, the midgut in virgin and mated females are morphologically and physiologically diverse [17, 20]. Mated females change defecation frequency, fecal pH and water content of the gut, and this mating responsiveness is crucial for regulating female post-mating nutrient absorption and egg production [17, 19, 20]. As food passes through the guts of mated females more slowly, this allows more time for nutrient absorption and thus more concentrated excreta [20]. The mated *D. melanogaster* female midgut also can accelerate gametogenesis by releasing enteroendocrine cells (EECs)-derived Neuropeptide F [17, 18]. Besides, genes involved in fatty acid metabolism are up-regulated in enterocytes (ECs) after mating in *D. melanogaster*, which may facilitate fecundity in females [17]. Post-mating transcriptomes in *Anopheles coluzzii* female midguts also showed that sugar transport, metabolism, and innate immune response genes were expressed inductively [26].

During mating, males transfer seminal fluid proteins that trigger the switch between virgin and mated female states. The transition involves a series of molecular, morphological, behavioral and physiological changes, and occurs in rapid and sustained phases [27–31]. Short-term post-mating responses occur during the first 24 h, while long-term post-mating responses can last up to two weeks after mating [27–30, 32]. The female post-mating response includes increased egg production and food intake, changes in food preference, decreased receptivity to remating, and diminished immune response [10–13, 28, 29, 33–38]. Male-derived Sex Peptide (SP), actings through Sex Peptide receptor (SPR) neurons in the female reproductive tract, has been connected to increases in intestinal transit time and stimulation of Neuropeptide F release from EECs in *D. melanogaster* midgut, and thus enhanced nutrition and fecundity [18, 20, 39, 40]. Mating significantly facilitates intestine growth specifically in females and enhances reproductive output as a result of juvenile hormone and ecdysone promoted ISC proliferation [17, 19, 22]. Despite the important connection between nutrition, gut physiology, and mating, little is known about the integrative and coordinated process involving numerous transcriptional changes triggered by mating in the female midgut and which processes are modulated to adjust midgut size and digestion to the demands of egg production.

The spotted wing *Drosophila*, *Drosophila suzukii* (Matsumura), is a global devastating and invasive agricultural pest that invaded Europe and the Americas. It causes severe economic loss due to damage to a wide variety of fruit crops such as waxberry, blueberries, strawberries, peaches, cherries, persimmon, and grapes. *D. suzukii* poses a huge threat to commercial soft fruit production and security due to its polyphagy, adaptability and robust fecundity [41–47]. Unlike the majority of *Drosophila* species, such as *D. melanogaster*, who oviposit on overripe fruit, *D. suzukii* lays eggs in healthy and undamaged ripening fruit, destroying crops through the rot and abscission of fruits. The infestation of *D. suzukii* female to ripe fruits is facilitated by the presence of a sclerotized and serrated ovipositor that enables piercing intact fruit skin and deposition of eggs into ripe fruits [44, 48, 49]. Chemical insecticides are currently considered as the most effective tool to control *D. suzukii*. However, the increase in resistance to commonly applied chemical insecticides necessitates the development of environment friendly pest management strategies [41, 50, 51]. Population replacement control strategies that utilize genetically modified pests show promise for integrated pest control, and these approaches are dependent on successful mating and reproduction [52–54]. Establishment of a positive energy homeostasis may be particularly important to *D. suzukii* that involves rapid production of large numbers of progeny, and the intestine plasticity may play significant role in this process during reproduction. Thus, identification of genes driven by mating in the *D. suzukii* female midgut will provide important insights for the development of novel approaches to control this pest by targeting the reproduction.

In the present study, the transcriptome of virgin and mated female *D. suzukii* midguts were sequenced using the Illumina HiSeq 2500 system. Furthermore, analysis of the differentially expressed genes between virgin and mated female midgut was carried out to identify the potential genes that respond to mating. The expression levels of the genes involved in the post-mating response at different time points were analyzed by qRT-PCR.

## Results

### Sequencing and assembly of virgin and mated female midgut transcriptomes

**Fig. 1:**
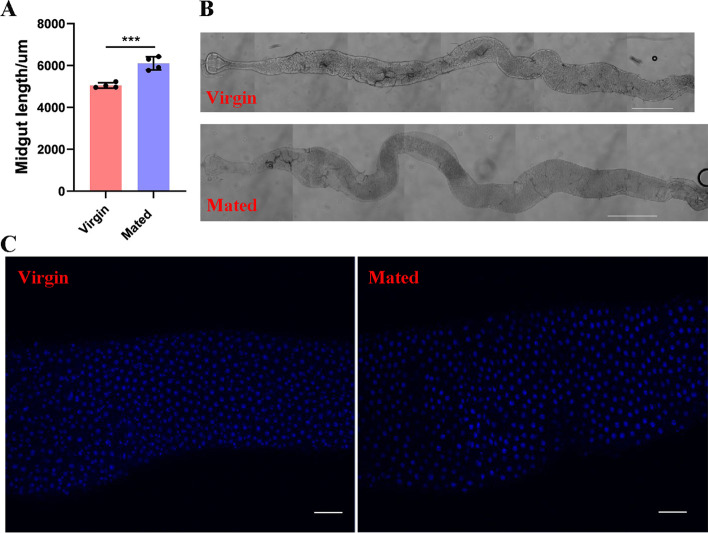
Post-mating change in midgut two days after mating in female *D. suzukii*. **A** Midgut length quantifications. The unit of length is um. Error bars indicate the SEM of three independent biological replicates and asterisks (***) indicate the statistically significant differences (*P* < 0.001) between virgin and mated female midgut based on Student’ s t-test. **B** Representative images of virgin and mated female midgut phenotypes. The scale bar is 500 um. **c** Changes in midgut cell proliferation revealed by DAPI staining. The scale bar is 20 um. The region of the midgut is anterior midgut

**Fig. 2:**
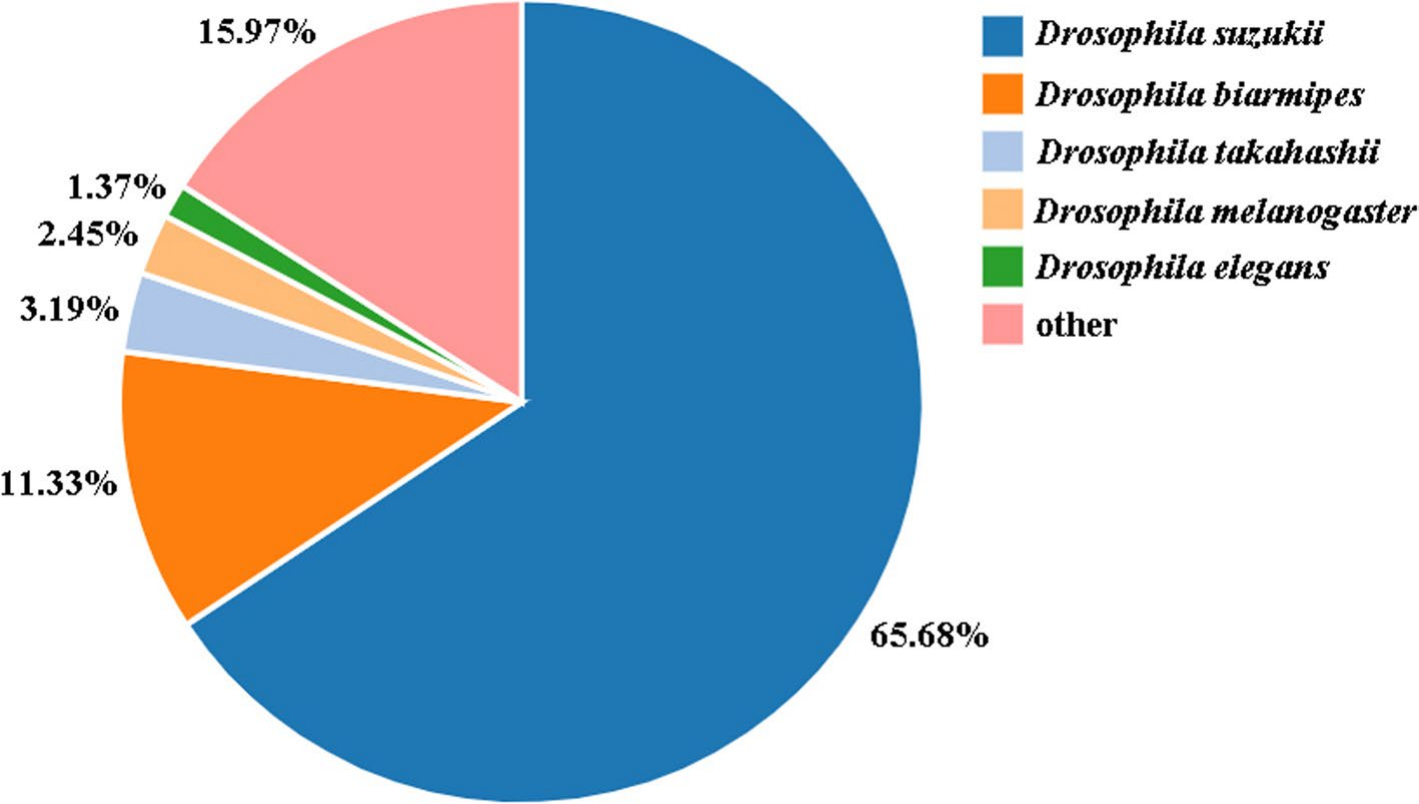
Species distribution of unigenes in the virgin and mated female midgut transcriptomes of *D. suzukii*

First, we examined the changes in midgut lengths at 1 d, 2 d and 3 d after mating and the midgut length was significantly longer than that of virgin controls at each time point in female *D. suzukii* (*P* < 0.001) (Fig. 1A and 1B and Figure S1). The average midgut length was 5614.25, 6102.75, 5529 um in the mated female midgut compared to the the average midgut length of 4406.5, 5048, 4612.25 um from the virgin controls at 1 d, 2 d and 3 d after mating respectively (Fig. 1A and Figure S1). The visibly longer and larger midgut phenotypes were observed under brightfield conditions at each time point after mating (Fig. 1B and Figure S1). This midgut enlargement in female *D. suzukii* is in accordance with the time frame of SP-mediated post-mating responses, such as increased egg production and reduced receptivity to remating, which persists for ten days [28, 33]. We also found that mating increases the number of intestinal cells significantly and leads to a visibly larger midgut diameter as revealed by DAPI stainings in female *D. suzukii* (Fig. 1C and Figure S1 and S2). To ascertain the impact of mating on digestive physiology, we characterized the transcriptome of whole midguts of virgin females, and females mated to males at 2 d post-mating. Transcriptome libraries of the virgin and mated female midguts were constructed and sequenced in the Illumina platform using paired-end sequencing. This generated a total of 262.92 million reads with high sequence quality (BioProject accession number: PRJNA827258). After removing low-quality reads, virgin female midgut libraries generated 43.13, 42.96 and 43.09 million clean reads while mated female midgut libraries generated 43.08, 43.18 and 43.00 million clean reads. Among these clean reads, 22.47–23.40 million (52.16%-54.20%), were mapped to genes in the whole genome sequence (WGS) of *D. suzukii* (Supplementary Table S1). The percentage of clean reads ranged from 98.03% to 98.43% in virgin female midgut libraries and 98.13% to 98.53% in mated female midgut libraries (Figure S3). The filtered sequence reads from all samples were assembled and produced 17,452 unigenes, which the total length, mean length, N50 and GC contents were 25,611,966 bp, 1,467 bp, 2,448 bp and 50.27%, respectively (Table 1). Gene sequences were annotated by searching the nonredundant NCBI protein database using BLASTX. A total of 15,275 unigenes (87.53%) were matched to known genes, among which 12,609 unigenes had the complete coding sequence (CDS) and 1,730 unigenes encoding transcription factor were predicted (Supplementary Table S2). Most of these sequences (84.03%) showed strong similarity to those of *Drosophila* species. Amongst them, 65.68% of these sequences best matched sequences from *D. suzukii*, followed by *D. biarmipes* (11.33%), *D. takahashii* (3.19%), *D. melanogaster* (2.45%), *D. elegans* (1.37%), and other insect species (15.97%) (Fig. 2). The average unigene size was 1,467 bp with lengths ranging from 200 to 25,224 bp (Table 1). There were 8,415, 4,299 and 2,232 genes whose length was larger than 1,000, 2,000, and 3,000 bp, respectively (Table 1 and Figure S4). The total analysis of transcriptome sequences suggested that the assembly quality was high and the data accuracy was reliable in *D. suzukii*.

**Table 1:** Summary of the virgin and mated *D. suzukii* female midgut transcriptomes

Total number of Unigene	17,452
Total Length (bp)	25,611,966
Mean Length (bp)	1,467
N50	2,448
N70	1,564
N90	672
GC (%)	50.27
Number of transcripts > 1 Kb	8,415
Number of transcripts > 2 Kb	4,299
Number of transcripts > 3 Kb	2,232

### Comparison of gene expression profiles in virgin and mated female midgut

**Fig. 3:**
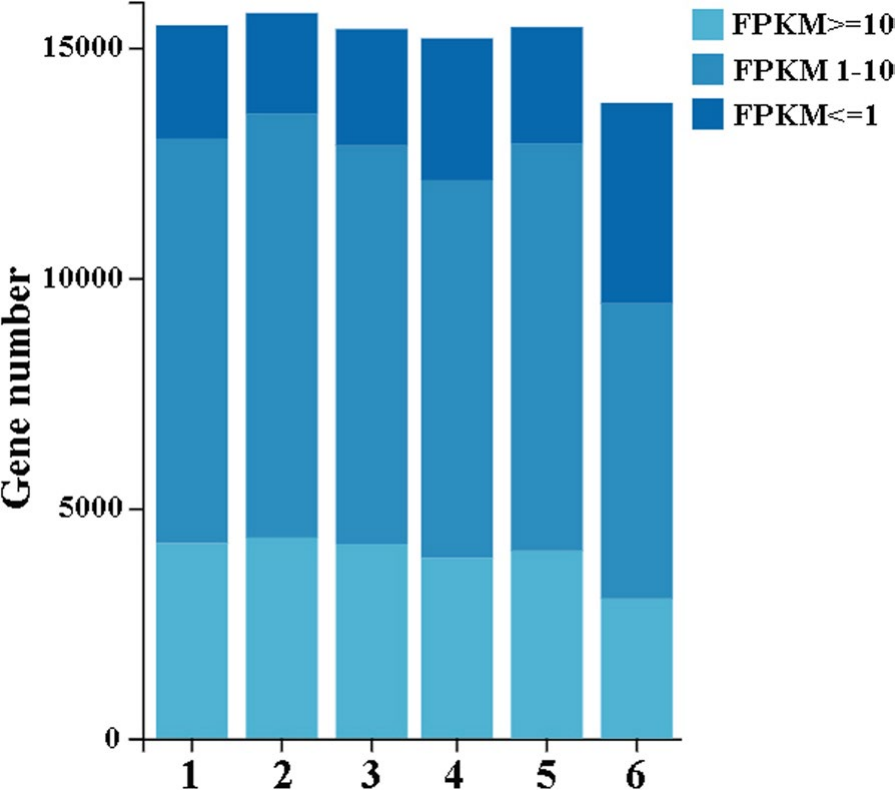
Fragments per kilobase of transcript per million mapped reads (FPKM) distribution of the *D. suzukii* virgin and mated female midgut transcriptomes. 1, 2, 3 represent the virgin female midgut samples and 4, 5, 6 represent the mated female midgut samples

**Fig. 4:**
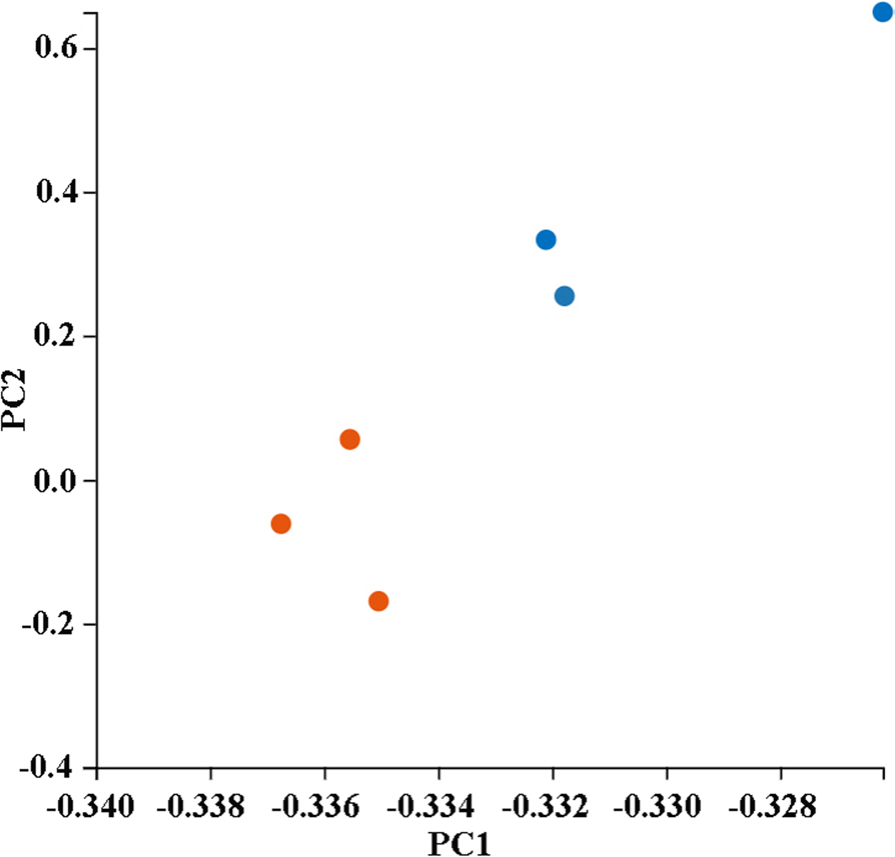
Principal component analysis of the six transcriptome samples. Samples are virgin female midgut (red) and mated female midgut (blue)

To assess the relative expression level of genes in the *D. suzukii* virgin and mated female midgut transcriptomes, we normalized the gene read counts by transforming them into Fragments Per Kilobase of transcript per Million mapped reads (FPKM). A broad extent of gene expression levels from less than 1 FPKM to 62,092 FPKM were obtained (Supplementary Table S2). 13.85% to 16.45% of the genes had a low expression level (FPKM <  = 1), 56.15% to 58.50% of the genes had a moderate expression level (FPKM 1–10), and 27.37% to 27.65% exhibited a high expression level (FPKM >  = 10) in the virgin female midgut libraries. While 16.32% to 31.58% of the genes had a low expression level (FPKM <  = 1), 46.46% to 57.34% of the genes had a moderate expression level (FPKM 1–10), and 21.96% to 26.34% exhibited a high expression level (FPKM >  = 10) in the mated female midgut libraries (Fig. 3). Principal components analysis of all six samples showed that both virgin and mated female midgut samples clustered together with their respective replicates (Fig. 4).

**Fig. 5:**
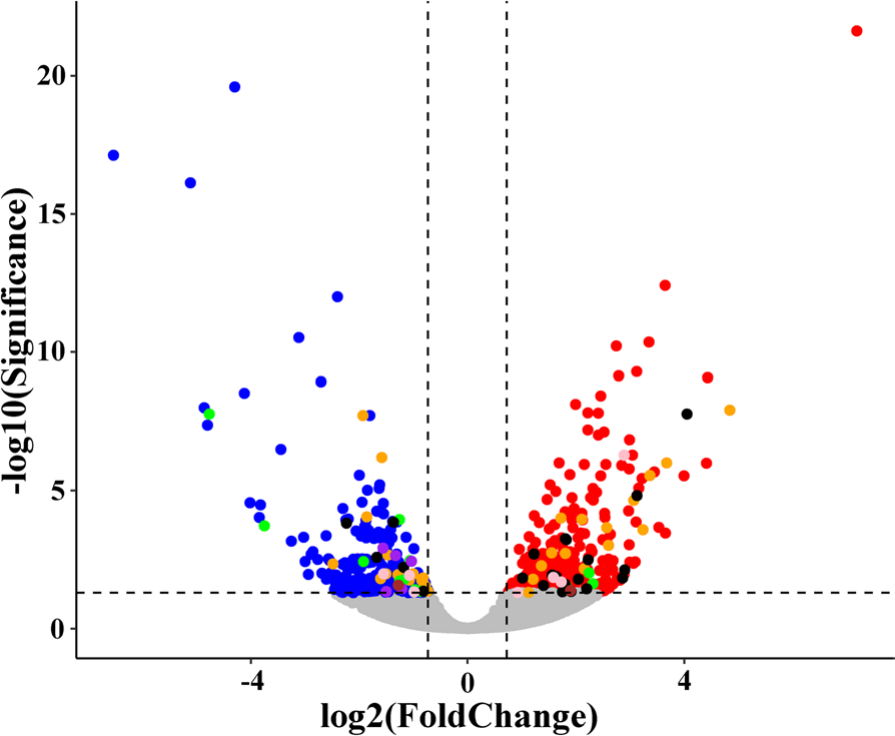
Volcano plot showing differentially expressed unigenes (|log2(FoldChange)|> 0 and padj < 0.05) in virgin and mated female midgut transcriptomes. Genes shown on top right corner are up-regulated in virgin female midgut and genes shown on top left corner are up-regulated in mated female midgut. The orange, dark and green dots indicate the genes involved in carbohydrate metabolism, lipid metabolism and amino acid metabolism, respectively. The pink dots indicate the genes involved in carbohydrate metabolism and lipid metabolism simultaneously. The violet dots indicate the genes involved in carbohydrate metabolism and amino acid metabolism simultaneously. The brown dots indicate the genes involved in carbohydrate metabolism, lipid metabolism and amino acid metabolism simultaneously

**Fig. 6:**
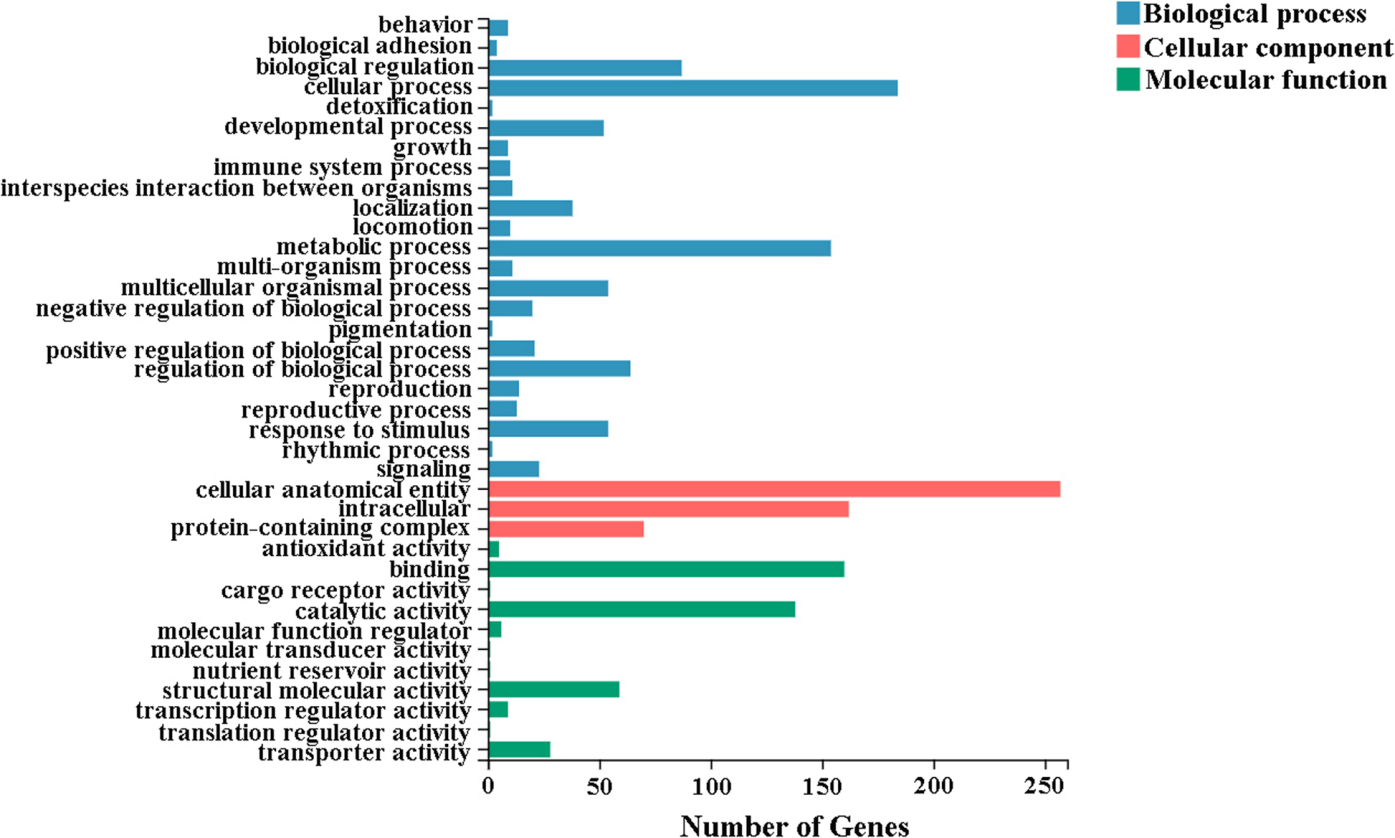
Gene Ontology (GO) significant enrichment analysis for differentially expressed genes (DEGs) between virgin and mated female midgut transcriptomes of *D. suzukii*

**Fig. 7:**
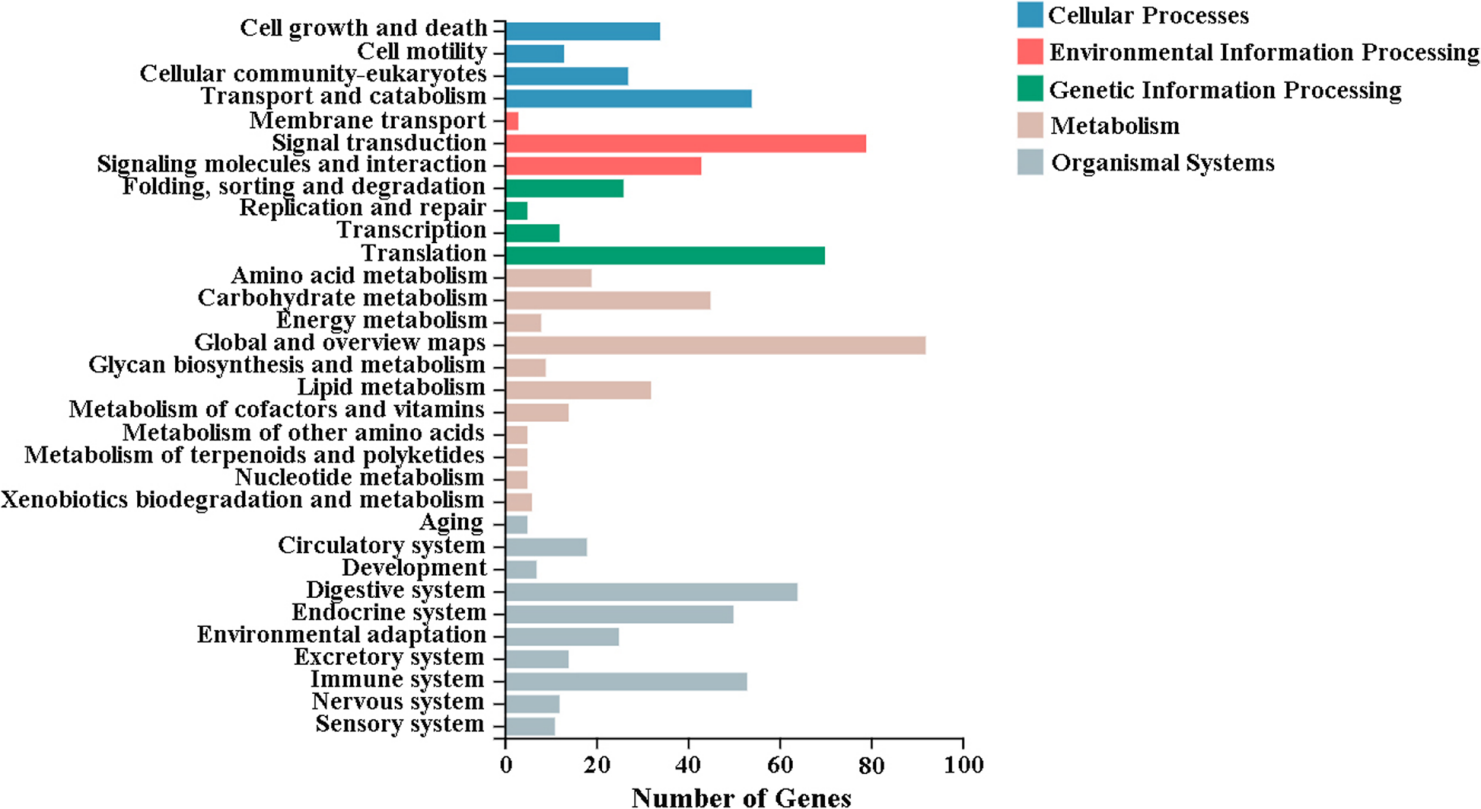
Kyoto Encyclopedia of Genes and Genomes (KEGG) significant enrichment analysis for differentially expressed genes (DEGs) between virgin and mated female midgut transcriptomes of *D. suzukii*

We further identified differentially expressed genes (DEGs) between virgin and mated female midgut. At a setting of *p* < 0.05, 652 DEGs were identified. 400 DEGs exhibited relatively higher expression levels in virgin female midgut than mated female midgut, and 252 DEGs showed relatively higher expression levels in mated female midgut than virgin female midgut (Fig. 5 and Supplementary Table S3). We then analyzed the functions of DEGs based on gene ontology (GO) classification. 652 DEGs were characterized into three groups: 236, 263 and 299 DEGs categorised into biological processes, cellular components, and molecular function, respectively (Fig. 6 and Supplementary Table S4). The largest representations were in cellular processes and metabolic processes (biological processes), cellular anatomical entity and intracellular (cellular component) and binding and catalytic activity (molecular function). In addition, enrichment comparisons showed that the cellular process, metabolic process, biological regulation, response to stimulus, developmental process and immune system process were included during mating in the biological processes (Fig. 6). Furthermore, enrichment analysis of Kyoto Encyclopedia of Genes and Genomes (KEGG) pathways for the DEGs was also performed (Fig. 7 and Supplementary Table S5). The results showed that metabolism pathways were the main groups, including carbohydrate metabolism, lipid metabolism, amino acid metabolism, metabolism of cofactors and vitamins, glycan biosynthesis and metabolism, and energy metabolism (Fig. 7).

### Transcriptional metabolism and immune changes of post-mating response in female midgut

**Fig. 8:**
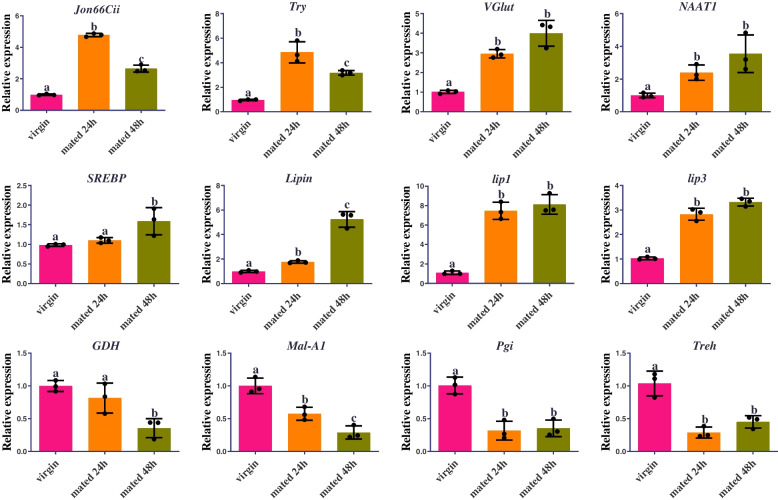
The effect of mating on the expression levels of metabolism-related genes was detected by qRT-PCR in the midgut of *D. suzukii* females. RNA was extracted from the midgut of mature vigin females as well as mated females at 24, 48 h after mating. Error bars indicate the SEM of three independent biological replicates and various letters represent statistically significant differences of the expression level of genes (*p* < 0.05, Student’ s t-test)

**Fig. 9:**
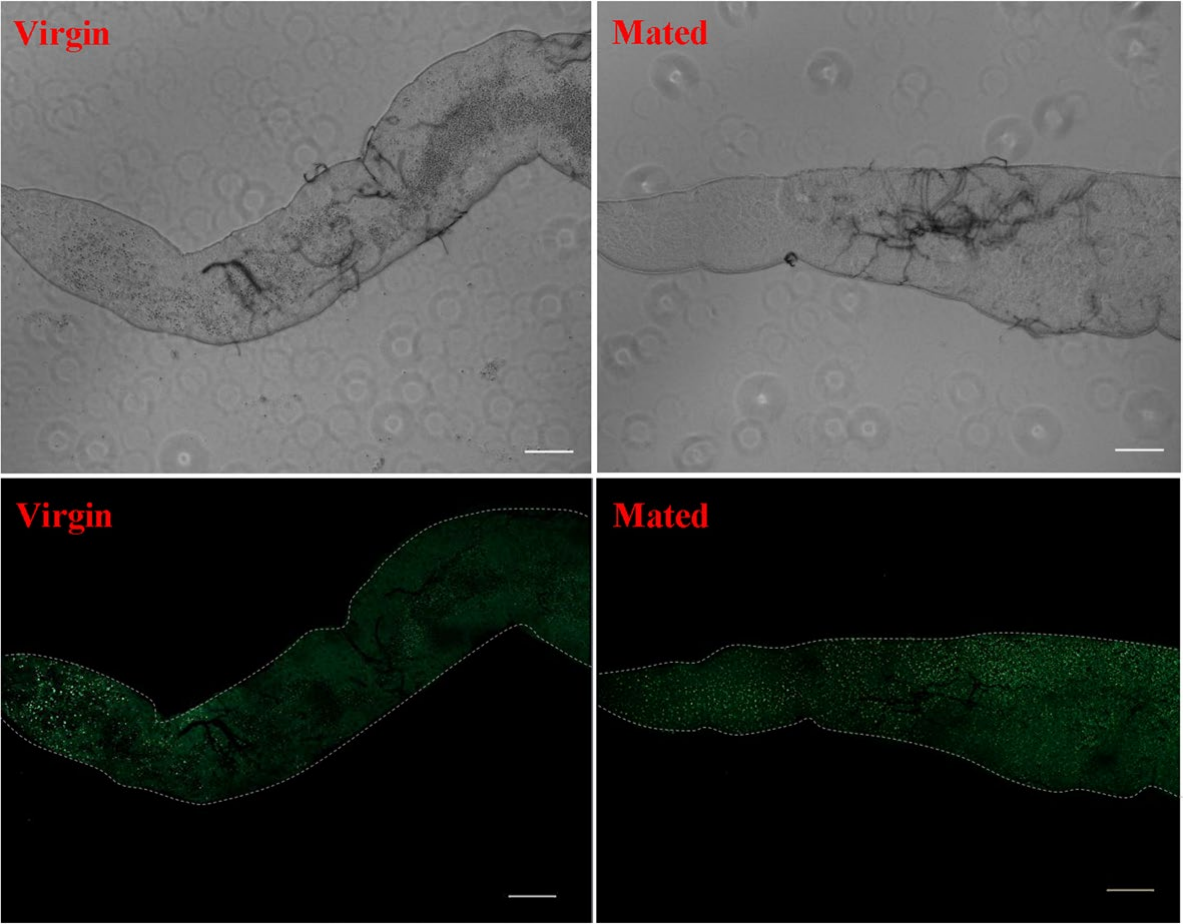
Mating increases neutral lipid content revealed by Bodipy staining in the virgin and mated female midgut of *D. suzukii*. The scale bar is 20 um. The region of the midgut is anterior midgut

To identify the influence of mating on the dynamic responses in female midgut, we sequenced the transcriptome of virgin female midguts and 2 d post-mating female midguts. Differential expression analysis disclosed 652 genes were differentially expressed in the midgut between virgin females and 2 d post-mating females (Supplementary Table S3), among which 252 and 400 genes were up-regulated and down-regulated respectively. We identified the up-regulated protein and lipid metabolism genes and down-regulated carbohydrate metabolism genes upon mating (Supplementary Table S3). To further validate numerous metabolism-related genes with respect to expression at different time points after mating, they were analyzed by quantitative Real-Time PCR (qRT-PCR). The results showed that the Jonah family of proteases, *Jonah 66Cii* (*Jon66Cii*) gene, the trypsin family protease *Trypsin* (*Try*) gene, the SLC family of transporters *Vesicular glutamate transporter* (*VGlut*) gene and the amino acid transporters *Nutrient Amino Acid Transporter 1* (*NAAT1*) gene were highly expressed in 24 and 48 h mated female midgut compared to the virgin control female midgut (Fig. 8). We also found up-regulation of genes involved in fatty acid and lipid metabolism (Fig. 8). For instance, *Sterol regulatory element binding protein* (*SREBP*), *Lipin*, *lipase 1* (*lip1*), *lipase 3* (*lip3*) were highly expressed in 24 and 48 h mated female midgut. Consistent with this up-regulation of lipid genes, female flies accumulated neutral lipid content revealed by Bodipy staining after 48 h mating in the midgut of *D. suzukii* (Fig. 9 and Figure S5). Meanwhile, we examined the down-regulated genes upon mating to males and found an enrichment of genes related to carbohydrate metabolism (Fig. 8). Carbohydrate metabolism genes such as *maltase A1* (*Mal-A1*), *Phosphoglucose isomerase* (*Pgi*), *glucose dehydrogenase* (*GDH*), *Trehalase* (*Treh*) were significantly down-regulated in 24 and 48 h mated female midgut compared to the virgin control. Altogether, these data suggest that mating induces a shift in midgut gene expression from carbohydrate metabolism to protein and lipid metabolism in *D. suzukii*.

**Fig. 10:**
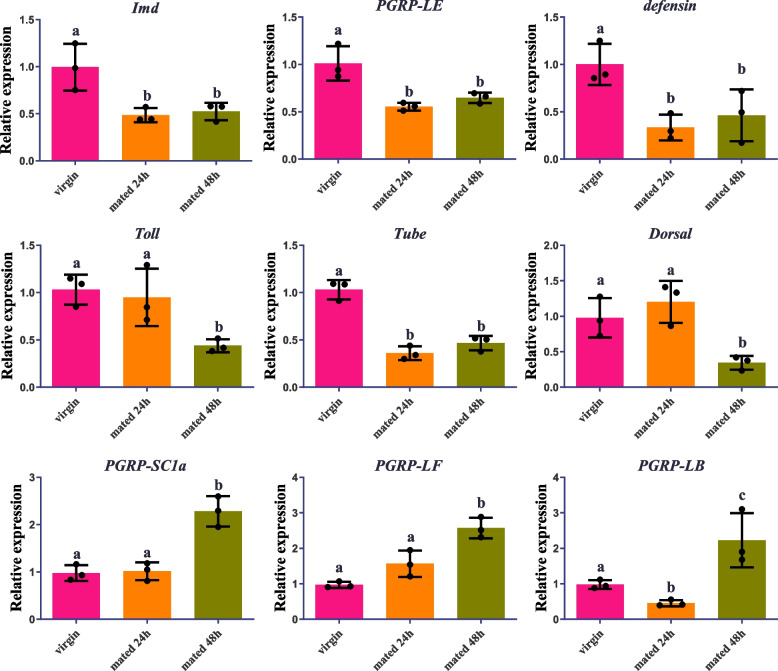
The effect of mating on the expression levels of immune-related genes was detected by qRT-PCR in the midgut of *D. suzukii* females. RNA was extracted from the midgut of mature vigin females as well as mated females at 24 h and 48 h after mating. Error bars indicate the SEM of three independent biological replicates and various letters represent statistically significant differences of the expression level of genes (*p* < 0.05, Student’ s t-test)

**Fig. 11:**
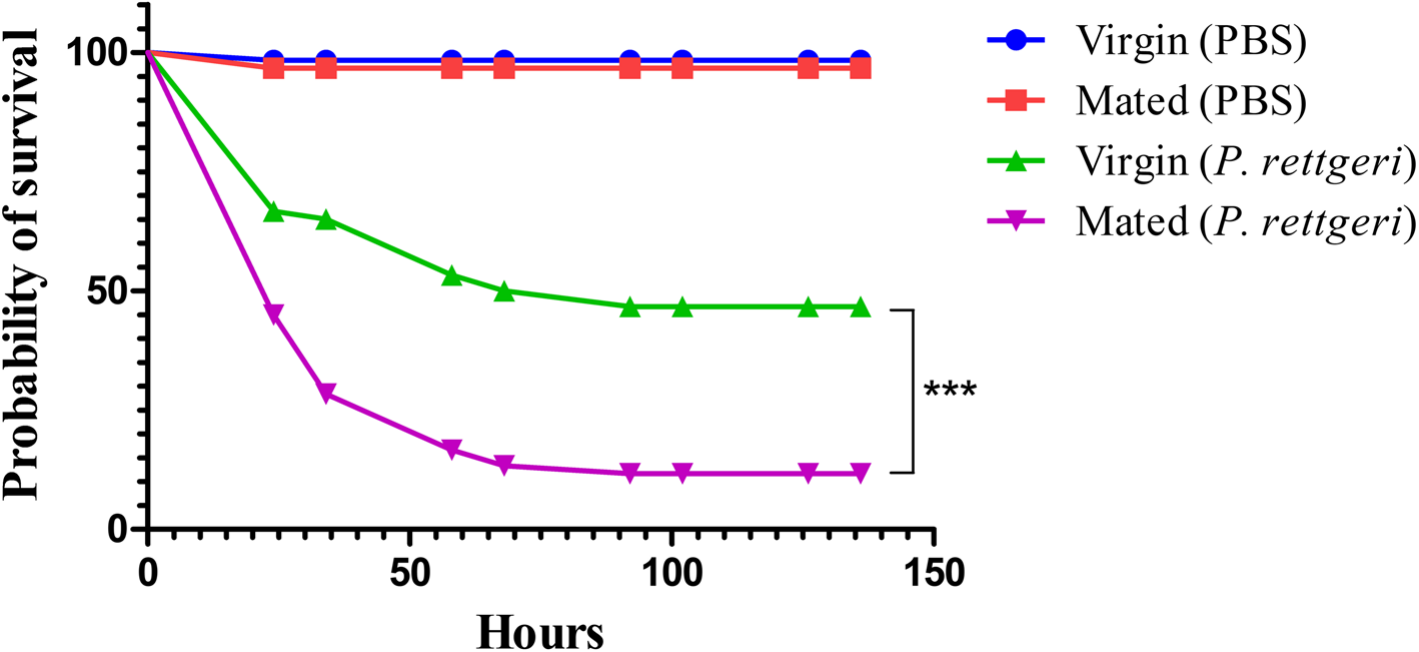
The effect of mating on immune defense after mating in *D. suzukii*. Survival over time of mated females was significantly lower than that of virgin controls when infected with *P. rettgeri*. Mortality was recorded each day for six days after infection, and survival curves were estimated using the Kaplan–Meier method. Control flies had negligible mortality over the course of the experiment regardless of mating treatment. ***p < 0.001

To analyze the mating-induced changes in immunity in *D. suzukii* virgin and mated female midgut transcriptomes, we identified multiple immune-related genes that were differentially expressed between virgin and mated female midgut (Supplementary Table S3). In order to explore the complicated post-mating response in immunity, the expression profiles of numerous immune-related genes in the female midgut at 24 and 48 h after mating were checked by qRT-PCR. The results showed that the positive regulators of the immune deficiency (IMD) pathway genes *Imd*, *peptidoglycan-recognition protein LE* (*PGRP-LE*), the antimicrobial peptides (AMPs) genes *defensin* (*def*), and the Toll signaling pathway genes *Toll*, *Tube*, *Dorsal* were down-regulated in the 24 and 48 h mated female midgut compared to the virgin control female midgut (Fig. 10). While the negative regulators of the IMD pathway genes *peptidoglycan-recognition protein SC1a* (*PGRP-SC1a*), *peptidoglycan-recognition protein LB* (*PGRP-LB*), *peptidoglycan-recognition protein LF* (*PGRP-LF*) were up-regulated in the mated female midgut (Fig. 10). All the qRT-PCR results of metabolism-related and immune-related genes were consistent with our deep sequencing data, which indicated that the current analysis is accurate. For functional evidence of immune suppression induced by mating in *D. suzukii*, we evaluated the differences in survivorship between virgin females and mated females that were all infected with the bacterium, *Providencia rettgeri*, 48 h after mating*.* The results showed that mated females had significantly lower survival rate than virgin females during the experimental process when infected with *P. rettgeri* in *D. suzukii*, while the virgin and mated females suffered similar low mortality rates when infected with PBS (Fig. 11). Higher levels of infection-induced mortality in mated females may indicated that there was a trade-off between reproduction and immunity in *D. suzukii*.

## Discussion

In this study, we performed a genome-wide transcriptomic analysis of the main digestive organ, the midgut, to investigate how mating status trigger shifts of gene expression in female midgut of *D. suzukii.* A total of 652 DEGs were identified, among which the expression pattern of 252 DEGs were up-regulated in mated female midgut and 400 DEGs were up-regulated in virgin female midgut. Differential expression profiles of transcripts involved in the metabolism and immune processes at different time points after mating were validated by qRT-PCR. As the first analysis of transcriptional mechanisms driven by mating in *D. suzukii* female midgut, this dataset not only provides a valuable resource necessary for a better understanding of the genetic basis of intestine plasticity during reproduction, but also provides abundant target genes for effective control of this agricultural pest.

Production of offspring requires significant energy investments and involves dramatic metabolic adaptations to enhanced energy demands in females. *D. melanogaster* increases egg production tenfold after mating and triggers multiple metabolic and behavioural adaptations such as food intake, digestion, and nutrient preference [10, 11, 17, 20, 28, 30, 55]. These shifts are largely induced by signals delivered by the male during copulation, in particular the seminal fluid molecule SP [28, 30]. In our study, we found that the midgut enlargement is detected at 1 d, 2 d and 3 d post-mating females of *D. suzukii* which is similar to the situation in *D. melanogaster* [17, 22]. This midgut enlargement is consistent with the time frame of SP-mediated post-mating responses, for instance increased egg production and reduced receptivity to remating which persists for 10 d [28, 33]. Besides, we have also identified SPR in the midgut with low expression, indicating the possibility that SP could act directly on the SPR in gut to stimulate post-mating gut growth. Across diverse studies of mating-regulation of gene expression in whole females or different female tissues such as midgut, spermathecae, head/brain, and reproductive tract, metabolic processes are the main shifts induced by mating in multiple insect species, for example, *Aedes aegypti*, *Anastatus disparis*, *Anopheles gambiae*, *Apis mellifera*, *Bactrocera dorsalis*, *Callosobruchus maculatus*, *D. melanogaster* [26, 56–68]. We observed down-regulation of genes involved in carbohydrate metabolism and up-regulation of genes involved in protein digestion and lipid metabolism in mated female midgut compared to virgin female midgut in *D. suzukii*, which show the similar mating-induced gene expression changes to *D. melanogaster* reported previously [22, 67–69]. *Pgi*, *Mal-A1*, *GDH* and *Treh* were down-regulation of genes involved in carbohydrate metabolism in mated *D. suzukii* female midgut, which are consistent with the observations of down-regulation of maltase genes in the whole-organism transcriptome of 3 to 5 d-old mated *D. melanogaster* females [69] and down-regulation of carbohydrate metabolic genes in *D. melanogaster* female abdomens at 3 h post-mating and in *D. melanogaster* female midgut at 2 days after mating [22, 70]. *Jon66Cii*, *Try*, *VGlut*, *NAAT1* were up-regulated genes involved in protein digestion and *SREBP, Lipin*, *lipase* were up-regulated genes involved in lipid metabolism in mated *D. suzukii* female midgut, which are consistent with the observations of post-mating up-regulation of Jonah family serine-type endopeptidase genes in *D. melanogaster* [68, 69] and up-regulation of several proteases in female and proteolysis-related genes in female abdomens 3 h after mating [67, 70]. The accumulation of neutral lipid content revealed by Bodipy staining after 48 h mating confirmed the up-regulation of lipid genes in mated female midgut of *D. suzukii*. We detected up-regulation of *SREBP* in the midgut after mating by qRT-PCR, which is consistent with the observation that the expression of fatty acid metabolic genes is induced upon mating [17, 22]. The lipid biosynthesis pathway is significantly up-regulated by mating in *A. ludens* and genes involved in fatty acid synthesis are up-regulated by mating in both *A. ludens* and *Anastatus disparis* [71, 72]. We also found down-regulation of *GstS1* which is involved in detoxification, and this mating induced down-regulation could affect the capacity of the female to cope with toxic dietary foods and oxidative stress [22, 73]. All these results demonstrated that mating could induce an increase in protein and lipid digestion in female insects, which is consistent with increased protein and lipid food intake after mating, which is necessary for yolk protein production and female fecundity [9, 74]. The up-regulation of protein and lipid metabolic genes and the coincident down-regulation of carbohydrate metabolic genes in mated *D. suzukii* female midgut may reflect the fact that females alter digestive parameters to adapt to new nutritional demands.

Reproduction and immunity are physiologically and energetically fastidious courses and the trade-off between these two crucial processes exists in numerous insects [38]. The trade-off between reproduction and immunity are driven by resource-allocation. The more biological resources insects invest in reproductive capacity, the lower the immune function, and vice versa. Indeed, many studies have documented trade-offs between reproduction and immunity in a diversity of insects. In the ground cricket, *Allonemobius socius*, reduced hemocyte number, encapsulation ability and lytic activity with increasing mating effort, results in an increased mortality rate of both sexes [75]. Increasing mating success led to a reduction in phenoloxidase activity and consequent immune suppression in mealworm beetles, *Tenebrio molitor*, and wood ants, *Formica paralugubris* [76, 77]. Besides, sperm viability is negatively correlated with immunity such as encapsulation ability and lysozyme in leaf-cutting ant queens and honey bee queens [78, 79], and this negative relationship between sperm viability and immune function is widespread in various male insects [80–85]. In our study, the positive regulators of the IMD pathway genes *Imd*, *PGRP-LE*, the AMPs genes *def*, and the Toll signaling pathway genes *Toll*, *Tube*, *Dorsal* were down-regulated in mated *D. suzukii* female midgut. While the negative regulators of the IMD pathway genes *PGRP-SC1a*, *PGRP-LB*, *PGRP-LF* were up-regulated in mated female midgut at different time points. All these results showed that mating induces a reduction of immunity in *D. suzukii* female midgut at 24 h and 48 h post-mating. The expression level of AMP gene *def* is up-regulated after mating in *Ae. aegypti*, *Atta colombica*, *B. dorsalis*, *Ceratitis capitata*, *D. melanogaster*, and *Lasius niger* [56, 57, 67, 86–88], and is down-regulated after mating in *A. mellifera* [62]. However, this is different from the results of the previous report on *C. capitata*, which revealed a large reduction in *def* expression after mating in the female abdomen [87]. Mating also reduces the survival rate of female *D. melanogaster* under all kinds of pathogenic infections, and mated females have higher pathogen loads and reduced expression level of AMP genes after pathogenic infection [86, 89, 90]. The expression level of *def* and other AMP genes were down-regulated and up-regulated in mated females compared with virgin females at 12 h and 24 h post-infection in *D. melanogaster*, respectively [86]. The expression differences among these genes indicated that mated females were vulnerable to adequate defence against bacterial infection than virgin females in *D. melanogaster* [90]. More studies are needed to elucidate the complex relationship between the expression level of AMP genes and female immune response at different post-infection time points after mating. We have discovered that mating reduces survivorship after infection in *D. suzukii* similar to the studies described previously [38, 86, 89]. Interestingly, some studies reported that the expression level of AMP genes were induced by mating in different tissues [67, 91–94], which conflict with results showing that mating reduces female resistance to infection in *D. melanogaster*. However, this up-regulation of AMP genes were mainly confined to the reproductive tract [93, 94], and this tissue-specific up-regulation may be a result of a regional defence against sexually transfered infection [95, 96] that may barely affect the immune process in defence of systemic infection. A previous study has revealed that juvenile hormone (JH) prevented autoimmunity in *D. melanogaster* reproductive tissues by suppressing immune signaling to support reproductive output [97]. JH also increased reproductive output via raised lipid metabolism [17], and sterile *D. melanogaster* females were resistant to the impact of mating on immunity [86]. Besides, ecdysone signalling promoted intestinal growth, particularly in mated *D. melanogaster* females, and facilitated fecundity [19]. Thus, more work should address the exploration of the complicated relationships among hormone signalling, immunity and reproduction in mated female insects in the future.

## Conclusion

This study demonstrates post-mating modulation at the transcriptional level of genes involved in the midgut of *D. suzukii*, a destructive and invasive soft fruit pest. Mating causes a shift in the transcriptome of midgut, and the post-mating midgut increased transcription of genes involved in lipid and protein metabolism, while decreasing mRNA levels of carbohydrate metabolism genes and immune-related genes. All these shifts may help the female meet the energetic demands of egg production. Thus, the identification of genes between virgin and mated females midgut will not only be crucial to a better understanding of molecular research related to intestine plasticity during reproduction, but also provide abundant target genes for the development of effective and ecofriendly pest control strategies.

## Materials and methods

### Insect rearing

*D. suzukii* were fed on an artificial diet consisting of cornmeal, yeast, soy flour, maltose syrup and agar at Hunan Normal University (Changsha, China) and cultured at 25 °C under 12 h light: 12 h dark photoperiod [98]. Methyl 4-hydroxybenzoate was used as a preservative to prevent the overgrowth of fungi and bacteria. The diet has been optimized and has no negative fitness on *D. suzukii* development, survival and fecundity which is described in a previous study [99]. Newly emerged virgin females and males were collected and sorted separately. Mature females and males were kept in standard *Drosophila* vials together for mating (ten females and ten males per vial). Midguts were dissected from two days after female mating and at the same age as virgin females.

### Midgut length measurements and stainings

Virgin females and males were collected and single-pair matings were conducted three days after eclosion. Matings were monitored within 5–30 min and females who mated for less than 15 min were discarded. After mating, females were aged in groups of 10 in new food vials. Age-matched virgin female control groups were also maintained. Guts were dissected in phosphate-buffered saline (PBS) at the stated time points (1 d, 2 d,3 d after mating) and fixed with 4% paraformaldehyde solution in PBS for 60 min. Samples were stained with DAPI and Bodipy in PBS-0.01% Triton X-100 (1:50,000; Sigma Aldrich) and mounted on slides. All staining images were obtained using a Zeiss AxioImager M2 fluorescence inverted microscope. The whole midgut images were obtained under brightfield and analysed quantitatively using ImageJ. Brightfield images and staining images were loaded into ImageJ and the line tool used to outline the midguts. The region of interest (ROI) was analyzed using the polygon tool of ImageJ. Maximum projections were adjusted for levels and offsets and filtered to remove noise. The same parameters for scans were used within one experiment. The integrated density of fluorescence was quantified by multiplying their relative intensity and the area in the relevant channel. Quantification of cell numbers in the midgut of virgin and mated females was carried out by counting individual nuclei marked by the DAPI through a 40 × objective. The length of spline curve drawn down the midline was regarded as the midgut length.

### RNA extraction and transcriptome sequencing

Midguts of the same age virgin and mated females were collected with three independently biological replicates, and each replicate contained 60 midguts. Total RNA was extracted from midguts of *D. suzukii* using Trizol (Invitrogen, Carlsbad, CA, USA) according to manual instruction. The degradation and contamination of all RNA samples was checked on a 1.0% agarose gel, total RNA was qualified and quantified using a Nano Drop and Agilent 2100 bioanalyzer (Thermo Fisher Scientific, MA, USA). The library construction for Illumina sequencing was conducted with a total amount of 1 μg RNA from midguts of virgin and mated females by using NEBNext® Ultra™ RNA Library Prep Kit for Illumina® (NEB, USA) according to the manufacturer’s instructions. Sample sequencing was conducted on the HiSeq4000 platform using paired-end (PE) technology.

### Transcript sequence analysis

Raw reads of fastq format was filtered with SOAPnuke (v1.5.2). Clean reads were generated by removing reads containing the sequencing adapter, reads containing ploy-N and low-quality reads from raw datasets. The reads whose low-quality base ratio (base quality less than or equal to 5) was more than 20% were removed. Meanwhile, Q20, Q30 and GC content of the clean data were calculated. All the subsequent analyses were based on the clean reads with high quality. The clean reads were mapped to the whole genome sequence (WGS) of *D. suzukii* using HISAT2 (v2.0.4) (https://www.ncbi.nlm.nih.gov/genome/?term=txid28584) [100, 101]. The discordant or unpaired alignments were discarded. The mapped reads were assembled by StringTie (v1.3.3b) in a reference-based method. The read numbers mapped to each gene were counted using HTSeq v0.9.1 and each gene expression level was further calculated by FPKM (Fragment Per Kilobase of exon model per million mapped reads) based on the gene length and the gene read counts. Due to the effect of sequencing depth and gene length for the read counts, both were taken into consideration, and the FPKM is currently the most generally utilized approach for evaluating gene expression levels [102, 103].

### Differential expression and GO, KEGG enrichment analysis

Differential expression analysis of virgin and mated female midgut libraries was accomplished utilizing the DESeq2 R package (1.16.1). The resulting *P*-values were corrected using the Benjamini and Hochberg’s method for managing the false discovery rate (FDR). Genes with an corrected *P*-value < 0.05 found by DESeq2 were appointed as differentially expressed genes (DEGs) [98]. Gene Ontology (GO) and Kyoto Encyclopedia of Genes and Genomes (KEGG) enrichment analysis of DEGs was performed by Phyper based on Hypergeometric test [104]. The significant levels of terms and pathways were corrected by *P*-value with a rigorous threshold (*P*-value < 0.05) by Bonferroni [98].

### Quantitative real-time PCR

The expression profiles of metabolism and immune-related genes were surveyed using quantitative Real-Time PCR (qRT-PCR). Total RNA was extracted using RNAiso Plus reagent (TaKaRa, Dalian, China) from 60 midguts per replicate, with 200 ng for each sample subjected to reverse transcription utilizing the PrimeScript™ RT Master Mix (TaKaRa, Dalian, China). The reverse transcription products were subsequent utilized for qRT-PCR using primers listed in Supplementary Table S6. qRT-PCR was implemented using the SYBR Green qRT-PCR mix following the manufacturer’s instructions in a real-time thermal cycler (Bio-Rad, Hercules, CA, USA) utilizing the cycling conditions: 95 °C for 10 min, 40 cycles of 95 °C for 15 s, 60 °C for 30 s and 72 °C for 30 s. Three biological and three technical replicates were accomplished with expression data analyzed by the 2^−△△Ct^ approach [105]. Dissociation curves were determined for each gene to confirm unique amplification. The expression of ribosomal protein 49 (*Rp49*) was utilized as an internal control to normalize gene expression.

### Survival assays

Virgin females and males were collected and housed in groups of 10 individuals and 30 individuals, respectively. Three days after eclosion, females and males were transferred to new food vials for the copulation and this process was monitored within 5–30 min to ensure all females were typically paired with males. The Gram-negative bacterium, *Providencia rettgeri*, was used for the infection experiments. CO_2_-anesthetized females at 48 h after mating and the same age virgin females were injected with *P. rettgeri* cultures (OD600 = 7) in the thorax using a pulled capillary needle mounted on a Nanoject II apparatus (Drummond Scientific). Females were placed into new food vials in groups of 10 immediately after infection. Females that did not recover from the injection within 8 h were removed as their death was due to experimental handling rather than infection. Survival was recorded daily for 6 days and the PBS-injected females rarely resulted in mortality (< 1%).

### Statistical analysis

All experiments were repeated in triplicate and analyzed using GraphPad Prism 5.0 (GraphPad Software, San Diego, CA, USA) or Microsoft Excel (Microsoft, Redmond, WA, USA) with results expressed as the mean ± SEM. Data was compared with either a two-way ANOVA, with subsequent *t* tests using Bonferroni post-tests for multiple comparisons, or with the Student’s *t* test. For all tests, differences were considered significant when *P* < 0.05.

## Supplementary Information

**Additional file 1: Supplementary Table S1. **Alignment statistics of the virginand mated *D. suzukii *female midgut RNA-Seq analysis.** Supplementary Table S2. **All genes description and FPKM value in *D.suzukii* virgin and mated female midguttranscriptomes.** Supplementary Table S3.** Differentially expressed genes in pairwise comparison in *D. suzukii* virgin and mated female midguttranscriptomes.** Supplementary Table S4. **GO classification of the differentially expressed genes in pairwise comparisonin *D. suzukii* virgin and mated femalemidguttranscriptomes. Three main categories, namely biological process (BP), cellularcomponent (CC), and molecular function (MF) were assigned to DEGs.** Supplementary Table S5. **KEGG pathwayenrichment analysis for differentially expressed genes in pairwise comparisonin *D. suzukii* virgin and mated femalemidguttranscriptomes.** Supplementary Table S6. **Primersused in our study.** Supplementary FigureS1. **Post-mating change in midgut length 1 and 3 days after mating in *D.suzukii*. Midgut length quantifications (A) and (D), representative imagesof virgin and mated female midgut phenotypes (B) and (E), changes in midgutrevealed by DAPI staining (C) and (F). The scale label is 500 um in picture Band E, and 20 um in picture C and F.** SupplementaryFigure S2. **Mating increases cell proliferation in female *D. suzukii *midgut.Error bars indicate the SEM of three independent biological replicates andasterisks (**) indicate the statistically significant differences (P < 0.01)between virgin and mated female midgut based on Student’ s t-test.**SupplementaryFigure S3. **Evaluation of sequence quality for the *D. suzukii *virgin and mated female midguttranscriptomes.** SupplementaryFigure S4. **Distribution of protein coding genes lengths in *D.suzukii* virgin and mated female midguttranscriptomes. The sizes of all protein coding genes were calculated.** SupplementaryFigure S5. **Mating increases neutrallipidcontent revealed by Bodipy staining in the whole virgin and mated female midgutof *D. suzukii*. The quantification is showed in the right. The scale bar is 500 um.

## Data Availability

All the RNA-sequencing reads have been deposited in the Sequence Read Archive (https://www.ncbi.nlm.nih.gov/bioproject/PRJNA827258) with the accession codes (BioProject accession number: PRJNA827258).

## Abbreviations

DEGs: Differentially expressed genes
RNA-seq: RNA sequencing
WGS: Whole genome sequence
FPKM: Fragments Per Kilobase of transcript per Million mapped reads
ISC: Intestinal stem cell
GO: Gene ontology
KEGG: Kyoto Encyclopedia of Genes and Genomes
EEC: Enteroendocrine cell
EC: Enterocytes
SP: Sex Peptide
SPR: Sex Peptide receptor
CDS: Complete coding sequence
PGRPs: Peptidoglycan recognition proteins
Imd: Immune deficiency
AMPs: Antimicrobial peptides
PBS: Phosphate-buffered saline
FDR: False discovery rate
Rp49: Ribosomal protein 49
