# Supplementary material for: Functional transcriptome analyses of Drosophila suzukii midgut reveal mating-dependent reproductive plasticity in females
Source: BMC Genomics. 2022 Oct 25;23:726. doi: 10.1186/s12864-022-08962-2 (PMC9598023; doi:10.1186/s12864-022-08962-2)
Supplement: Supplementary file 1 — Additional file 1: Supplementary Table S1. Alignment statistics of the virginand mated D. suzukii female midgut RNA-Seq analysis. Supplementary Table S2. All genes description and FPKM value in D.suzukii virgin and mated female midguttranscriptomes. Supplementary Table S3. Differentially expressed genes in pairwise comparison in D. suzukii virgin and mated female midguttranscriptomes. Supplementary Table S4. GO classification of the differentially expressed genes in pairwise comparisonin D. suzukii virgin and mated femalemidguttranscriptomes. Three main categories, namely biological process (BP), cellularcomponent (CC), and molecular function (MF) were assigned to DEGs. Supplementary Table S5. KEGG pathwayenrichment analysis for differentially expressed genes in pairwise comparisonin D. suzukii virgin and mated femalemidguttranscriptomes. Supplementary Table S6. Primersused in our study. Supplementary FigureS1. Post-mating change in midgut length 1 and 3 days after mating in D.suzukii. Midgut length quantifications (A) and (D), representative imagesof virgin and mated female midgut phenotypes (B) and (E), changes in midgutrevealed by DAPI staining (C) and (F). The scale label is 500 um in picture Band E, and 20 um in picture C and F. SupplementaryFigure S2. Mating increases cell proliferation in female D. suzukii midgut.Error bars indicate the SEM of three independent biological replicates andasterisks (**) indicate the statistically significant differences (P < 0.01)between virgin and mated female midgut based on Student’ s t-test.SupplementaryFigure S3. Evaluation of sequence quality for the D. suzukii virgin and mated female midguttranscriptomes. SupplementaryFigure S4. Distribution of protein coding genes lengths in D.suzukii virgin and mated female midguttranscriptomes. The sizes of all protein coding genes were calculated. SupplementaryFigure S5. Mating increases neutrallipidcontent revealed by Bodipy staining in the whole virgin and mated female midgutof D. s [file 12864_2022_8962_MOESM1_ESM.zip › Supplementary material/Supplementary legends.docx]

**Supplementary legends:**

**Supplementary Table S1** Alignment statistics of the virgin and mated *D. suzukii* female midgut RNA-Seq analysis.

**Supplementary Table S2** All genes description and FPKM value in *D. suzukii* virgin and mated female midgut transcriptomes.

**Supplementary Table S3** Differentially expressed genes in pairwise comparison in *D. suzukii* virgin and mated female midgut transcriptomes.

**Supplementary Table S4** GO classification of the differentially expressed genes in pairwise comparison in *D. suzukii* virgin and mated female midgut transcriptomes. Three main categories, namely biological process (BP), cellular component (CC), and molecular function (MF) were assigned to DEGs.

**Supplementary Table S5** KEGG pathway enrichment analysis for differentially expressed genes in pairwise comparison in *D. suzukii* virgin and mated female midgut transcriptomes.

**Supplementary Table S6** Primers used in our study.

**Supplementary Figure S1** Post-mating change in midgut length 1 and 3 days after mating in *D. suzukii*. Midgut length quantifications (A) and (D), representative images of virgin and mated female midgut phenotypes (B) and (E), changes in midgut revealed by DAPI staining (C) and (F). The scale label is 500 um in picture B and E, and 20 um in picture C and F.

**Supplementary Figure S2** Mating increases cell proliferation in female *D. suzukii* midgut. Error bars indicate the SEM of three independent biological replicates and asterisks (**) indicate the statistically significant differences (P < 0.01) between virgin and mated female midgut based on Student’ s t-test.

**Supplementary Figure S3** Evaluation of sequence quality for the *D. suzukii* virgin and mated female midgut transcriptomes.

**Supplementary Figure S4** Distribution of protein coding genes lengths in *D. suzukii* virgin and mated female midgut transcriptomes. The sizes of all protein coding genes were calculated.

**Supplementary Figure S5** Mating increases neutral lipid content revealed by Bodipy staining in the whole virgin and mated female midgut of *D. suzukii*. The quantification is showed in the right. The scale bar is 500 um.
